# The Ryerson Audio-Visual Database of Emotional Speech and Song (RAVDESS): A dynamic, multimodal set of facial and vocal expressions in North American English

**DOI:** 10.1371/journal.pone.0196391

**Published:** 2018-05-16

**Authors:** Steven R. Livingstone, Frank A. Russo

**Affiliations:** 1 Department of Psychology, Ryerson University, Toronto, Canada; 2 Department of Computer Science and Information Systems, University of Wisconsin-River Falls, Wisconsin, WI, United States of America; University of Pécs Medical School, HUNGARY

## Abstract

The RAVDESS is a validated multimodal database of emotional speech and song. The database is gender balanced consisting of 24 professional actors, vocalizing lexically-matched statements in a neutral North American accent. Speech includes calm, happy, sad, angry, fearful, surprise, and disgust expressions, and song contains calm, happy, sad, angry, and fearful emotions. Each expression is produced at two levels of emotional intensity, with an additional neutral expression. All conditions are available in face-and-voice, face-only, and voice-only formats. The set of 7356 recordings were each rated 10 times on emotional validity, intensity, and genuineness. Ratings were provided by 247 individuals who were characteristic of untrained research participants from North America. A further set of 72 participants provided test-retest data. High levels of emotional validity and test-retest intrarater reliability were reported. Corrected accuracy and composite "goodness" measures are presented to assist researchers in the selection of stimuli. All recordings are made freely available under a Creative Commons license and can be downloaded at https://doi.org/10.5281/zenodo.1188976.

## Introduction

The study of emotion has advanced rapidly over the last decade, driven by low-cost smart technologies and broad interest from researchers in neuroscience, psychology, psychiatry, audiology, and computer science. Integral to these studies is the availability of validated and reliable expressions of emotion. To meet these needs, a growing number of emotion stimulus sets have become available. Most sets contain either static facial expressions or voice recordings. Few contain audiovisual recordings of speakers in North American English. Clinically, there is growing recognition for the role of singing in understanding neurological disorders and facilitating rehabilitation. Yet there are few validated sets of sung emotional expression. To address these needs, we developed the RAVDESS, a large validated set of audiovisual speech and song in North American English. This paper describes the creation of the RAVDESS, and reports validity and reliability data based on ratings from healthy, adult participants.

### The importance of multimodal communication

A trend in emotion research has been the use of affective stimuli that depicts emotion in a single modality, primarily through facial expressions. However, in the natural world emotional communication is temporal and multimodal. Studies have highlighted the importance of multisensory integration when processing affective stimuli [1–13]. The absence of validated multimodal sets has motivated researchers to create their own multimodal stimuli [5, 7, 14–18]. Researchers have also created multimodal stimuli by combining two independent unimodal sets [19], or joining self-created stimuli with an existing unimodal set [20]. This ad hoc approach may complicate the comparison of findings across studies, as each set varies in features, technical quality, and expressive intensity. Thus, divergent findings may be partially attributable to variations in stimulus sets.

### The need for dynamic facial expressions

Normal conversation contains a variety of expressions, and faces are rarely, if ever, static. Yet most sets contain only static facial images [21–33]. There is now substantial evidence that facial movement facilitates affective processing [34–44]. Imaging studies have revealed that dynamic expressions evoke differential and enhanced patterns of neural activation relative to static expressions [45–47]. Electromyographic studies have shown that dynamic stimuli elicit larger mimicry responses in the facial muscles of observers than those elicited by static expressions [48, 49]. Thus, dynamic facial expressions may provide a more ecologically valid representation of emotion than static facial expressions.

### Distinguishing features of the RAVDESS

There are five distinguishing features of the RAVDESS that build on popular existing sets.

#### Scope

First, whereas many sets contain fewer than 200 clips [21–24, 26, 28, 30, 50], the RAVDESS contains 7356 clips. The factorial design of the RAVDESS is visualized in S1 and S2 Figs. To our knowledge, only three other sets contain over 1000 recordings of dynamic, multimodal conversation [51–53]. The RAVDESS consists of 24 professional actors, each performing 104 unique vocalizations with emotions that include: happy, sad, angry, fearful, surprise, disgust, calm, and neutral. Each recorded production of an actor is available in three modality formats: audio-visual (AV), video-only (VO), and audio-only (AO). This diversity may be useful in repeated measures designs, as imaging studies have shown that key brain structures habituate to repeated presentations of the same stimulus [54, 55]. A large corpus of recordings is also useful for researchers in machine learning. The validated database is particularly well suited to machine learning approaches involving supervised learning, such as emotion classifiers [56], as they provide a large set for training and testing different algorithms.

#### Emotional intensity

Second, all emotions have been performed at two levels of emotional intensity, normal and strong. To our knowledge, only two other sets provide a controlled manipulation of intensity [57, 58]. Intensity is one of the most salient aspects of emotion [59], and has a prominent role in several theories of emotion [60–64]; note, the terms ‘intensity’ and ‘activation’ have been used interchangeably in these works. In these models, intensity often forms one of several orthogonal axes in a multidimensional emotional space. Perceptually, intense facial and vocal expressions are identified more accurately than their less intense counterparts [65, 66]. Intense facial expressions are also identified more quickly than their less intense counterparts [67], and elicit stronger facial mimicry responses in observers [68]. Thus, intense displays may be useful when researchers seek clear, unambiguous emotional exemplars. In contrast, normal intensity expressions may be required when investigating subtle differences in emotional perception [69], or for researchers seeking portrayals similar to those found in everyday life.

#### Two baseline emotions

Third, the RAVDESS includes two baseline emotions, neutral and calm. Many studies incorporate a neutral or “no emotion” control condition. However, neutral expressions have produced mixed perceptual results [70], at times conveying a negative emotional valence [71]. Researchers have suggested that this may be due to uncertainty on the part of the performer as to how neutral should be conveyed [66]. To compensate for this a calm baseline condition has been included, which is perceptually like neutral, but may be perceived as having a mild positive valence. To our knowledge, the calm expression is not contained in any other set of dynamic conversational expressions, and is present in one static facial image set [29].

#### North-American actors and raters

Fourth, the RAVDESS provides audiovisual recordings of vocal communication in North American English. Three existing sets present validated, audiovisual expressions of vocal emotional communication: the Geneva Multimodal Emotion Portrayal (GEMEP) [57, 72], CREMA-D [52], and MSP-IMPROV [51]. The GEMEP consists of 10 French-speaking actors, expressing a range of emotions, at three levels of intensity, in three vocal conditions (improvised sentences, pseudo-speech, and nonverbal affect bursts). The GEMEP is an exemplary and detailed set. However, the geographic origin of the GEMEP may pose issues for researchers in North America.

The pseudo-speech and improvised sentences of the GEMEP are spoken with a French accent. This may be unsuitable for researchers who require vocal content from the same geographic region or language as their participants. The facial expressions of the GEMEP actors may also signal a different geographical region due to the presence of nonverbal accents [73]. These accents can be subtle enough to distinguish cultures that share a common language, such as Australia and the United States [74]. Finally, the GEMEP stimuli have been validated by individuals of the same cultural region as the GEMEP actors. However, there is significant evidence of an ‘in-group’ advantage for emotional recognition, where accuracy is higher for emotions expressed and recognized by members of the same cultural group [75]. Reported accuracy rates of the GEMEP stimuli may differ when used with North American participants.

The CREMA-D consists of 91 English-speaking actors, expressing six spoken emotions. One sentence was produced at three levels of intensity, the other 11 sentences with unspecified intensity. This extensive set of 7442 recordings was validated by 2443 raters using crowd-sourced participants (Survey Sampling International) in an Internet-presented format, providing approximately 10 ratings per clip.

The MSP-IMPROV consists of 12 English-speaking actors, expressing four spoken emotions in a novel dyadic-conversational scenario. Fifteen sentences were produced with unspecified intensity. This large set of 7818 recordings was validated by over 50000 raters using crowd-sourced participants (Amazon Mechanical Turk) in an Internet-presented format. A core set of 652 clips were each rated 28 times, while remaining clips were each rated approximately 5 times.

A fourth set, the eNTERFACE’05, also provides audiovisual expressions of vocal emotional communication [76]. The set consists of 42 English-speaking lay-expressers from different countries, expressing six emotions in scenario-elicited format. Five distinct sentences for each emotion were produced with unspecified intensity. Recordings were included based on the judgements of two trained investigators. However, no measures of accuracy or reliability were provided. As such, the set cannot be assessed and compared against the performance of the RAVDESS or other existing sets.

The RAVDESS was designed for researchers and participants located in North America. It consists of 24 English-speaking actors, drawn from the Toronto area of Ontario, Canada. The 319 raters chosen to evaluate the RAVDESS stimuli were drawn from the same region.

#### Singing corpus

The final distinctive feature of the RAVDESS is that it includes a validated corpus of emotional song. Music is increasingly being used to understand cognitive and neural function in healthy and disordered populations [77–83]. Music has been used as a nonpharmacological treatment in the rehabilitation of neurological and motor disorders [84], including: cognitive recovery following stroke [77], mood improvement in depression [85], reduction of anxiety in obsessive compulsive disorder [86], recognition of speech emotion in children with cochlear implants [87], language function in aphasia [88], and motor rehabilitation in Parkinson’s disease [89, 90]. The RAVDESS offers clinical therapists a set of validated expressions of sung musical emotion from which to develop rehabilitative and diagnostic options. The RAVDESS is lexically-matched in song and speech. This feature may be beneficial for understanding processing differences in speech and song, or for examining disorders in which speech-music overlaps play a central role [91–93]. Specifically, the use of lexically matched utterances removes a confounding factor in studies seeking to compare speech with song or music [94, 95].

### Creation and validation of a new multimodal set

In the following sections, we present validation and reliability data in support of the RAVDESS. For the validation task, 247 participants each rated a subset of the 7356 files. For the reliability task, a further 72 participants provided intra-participant test-retest data. Validation was achieved by asking participants to label the expressed emotion. In several existing databases of facial emotion, an alternate rating method of validation has been implemented using a limited number of highly-trained participants to identify specific facial muscle contractions, or action units, which are then used to indicate a target emotion [96–98]. These systems were developed for nonverbal expressions of emotion, which involve relatively still faces. In contrast, vocal production involves significant orofacial movement, where movements tied to lexical content interact with movements related to emotional expression [13]. Thus, traditional muscle coding systems are unsuitable for validating the RAVDESS.

The validity task presents measures of emotional accuracy, intensity, and genuineness for all stimuli. These data, presented in S1 Table, provide a granular view of the RAVDESS stimuli. To assist researchers in the selection of appropriate stimuli, we include a composite “goodness” score, see also [33]. Goodness scores range between 0 and 10, and are a weighted sum of mean accuracy, intensity, and genuineness measures. The equation is defined such that stimuli receiving higher measures of accuracy, intensity, and genuineness, are assigned higher goodness scores.

## Method

### Ethics declaration

The RAVDESS and validation experiment used human volunteers. Informed written consent was obtained prior to any experiment or recording from all participants. Facial images of several actors are displayed later in this manuscript. These individuals gave written informed consent, as outlined in the PLOS consent form, to publish these case details. Participants and data from participants were treated according to the Declaration of Helsinki. The recording methods of the database and the subsequent validation experiment were approved by the local ethics committee of Ryerson University, Canada.

### Development of the RAVDESS stimuli

#### Actors

Twenty-four professional actors, working in Toronto, Ontario, Canada were hired for stimulus creation (M = 26.0 years; SD = 3.75; age range = 21–33; 12 males and 12 females). Actors self-identified as Caucasian (N = 20), East-Asian (N = 2), and Mixed (N = 2, East-Asian Caucasian, and Black-Canadian First nations Caucasian). To be eligible, actors needed to have English as their first language, speak with a neutral North American accent, and to not possess any distinctive features (e.g., beards, facial tattoos, hair colorings, facial piercings). Participants were also required to identify text presented at 1.5 m distance without wearing glasses.

Professional actors were selected over lay expressers for several reasons. Studies have shown that actor portrayals of emotion are identified more readily than those of lay expressers [99]. While one recent study found that vocal expressions of actors are only marginally more accurate than those of lay-expressers [100], it is unknown if the same holds true for facial expressions or dynamic audio-visual expressions. A growing number of emotion sets have successfully used professional or trained actors [29, 32, 33, 50, 57]. As with the RAVDESS, the creation of FACS-posed expressions was not the goal of these sets. Finally, the use of trained individuals is common in psychological tasks, such as music performance [101]. Actors are often recruited for studies of emotional expression [95], as they have received extensive training on the realistic portrayal of emotion.

The Toronto accent is a good example of the Standard North American English commonly found in Hollywood movies. The most notable exception is what has come to be known as Canadian raising, whereby diphthongs are raised when occurring before a voiceless consonant. For example, the /aʊ/ found in “house” or “about” will be somewhat raised to /^ʊ/. Canadian raising can be found in most parts of Canada, as well as northeastern New England, the Pacific Northwest, and the Upper Midwest. Critically, this accent feature is not prominent in the Toronto region and it is not found in the RAVDESS stimulus statements.

#### Stimuli

Two neutral statements were used (“Kids are talking by the door”, “Dogs are sitting by the door”). Statements were seven syllables in length and were matched in word frequency and familiarity using the MRC psycholinguistic database [102]. For the singing trials, statements were associated with melodies that were sounded using piano MIDI tones of fixed acoustic intensity, consisting of six eighth notes (300 ms) and ending with a quarter note (600 ms). The tonality of melodies associated with each emotion was tailored to be consistent with emotional association [103, 104]. The melody associated with the positively valenced emotions calm and happy was in the major mode (F3, F3, A3, A3, F3, E3, F3). The melody associated with the negatively valenced emotions sad, angry, and fearful was in the minor mode (F3, F3, A^b^3, A^b^3, F3, E3, F3). The melody associated with neutral emotion did not contain the third scale degree (F3, F3, G3, G3, F3, E3, F3) and was designed to be ambiguous in terms of major or minor mode.

The perceived valence of song melodies was validated in a separate a perceptual task. Eight participants (5 female, 3 male, mean age = 27.4, SD = 9.2), from Ryerson University, Toronto volunteered to participate. Raters had varied amounts of private musical instruction (mean = 9.0 years, SD = 7.1). Participants were asked to rate the perceived valence of each of the three melodies (major-mode, neutral, minor-mode), using a 9-point valence scale from the self-assessment-manikin (SAM) [105]. Results confirmed that the major-mode melody (M = 7.88, SD = 1.13) was rated as more positive than the neutral melody (M = 5.13, SD = 1.55), which in turn was rated as more positive than the negative melody (M = 3.0, SD = 1.77).

The stimulus timeline consisted of three main epochs: Task presentation (4500 ms), Count-in (2400 ms), and Vocalization (4800 ms). In the Task presentation epoch, the statement and emotion to be produced by the vocalist were presented on screen as text for 4500 ms. In the song condition, the melody to be used by the vocalist was sounded (2400 ms) after the text had been on screen for 1000ms. The Count-in epoch presented a visual count-in timer (‘1’, ‘2’, ‘3’, ‘4’) at an interonset interval of 600ms. The start of the Vocalization epoch was signaled with a green circle that was displayed for 2400 ms. The stimulus timeline began with an auditory beep (500 ms) and 1000ms of silence, and ended with an auditory beep (500 ms). The total duration of the stimulus trial was 13700 ms.

#### Selection of emotions

Eight emotions were selected for speech: neutral, calm, happy, sad, angry, fearful, surprise, and disgust. Calm and neutral were selected as baseline conditions, while the remaining states constitute the set of six basic or fundamental emotions that are thought to be culturally universal [106]. The concept of primary emotions has a long history in science and philosophy [107–109], with modern proponents [110–112]. While the discrete model of emotion has been criticized [113–115], it is a practical choice in the creation and labelling of emotion sets. Consequently, these six emotion labels can be found in most existing sets [21, 24–27, 29–31, 50, 57, 116–119]. The categorization of surprise as a basic emotion has been questioned by some theorists [114], while others have argued for its inclusion as a primary emotion [112]. As the debate remains unsettled, and as surprise is included in many existing sets, surprise was included in the speech set of the RAVDESS.

For song, six emotions were selected: neutral, calm, happy, sad, angry, and fearful. These emotions were selected as they are representative of expressions often conveyed in music [104, 120, 121]. Surprise and disgust were not included as they are rarely expressed in music and exhibit poor rates of reliability in listener studies [122–124].

#### Emotional elicitation

The RAVDESS was created using induced emotional expressions. These expressions have been variously referred to as simulated, posed, portrayed, enacted, instructed, or “felt experience acting” [57, 125–127]. In this type of elicitation procedure, actors used trained techniques to induce the desired emotional state prior to expression.

In the RAVDESS, actors were told that they could use whatever techniques they were trained in to induce the desired state, such as method acting or Stanislavski’s emotional memory techniques [128]. Actors were told that they would be given as much time as was needed for them to enter the desired emotional state, and that once achieved, they would signal their readiness. It was emphasized that actors were to provide genuine expressions of emotion, and that they were to be physically and mentally experiencing the intended emotion. Actors were told not to “indicate”—a pejorative acting term that refers to a non-truthful performance [129].

#### Procedure and design

**Fig 1:**
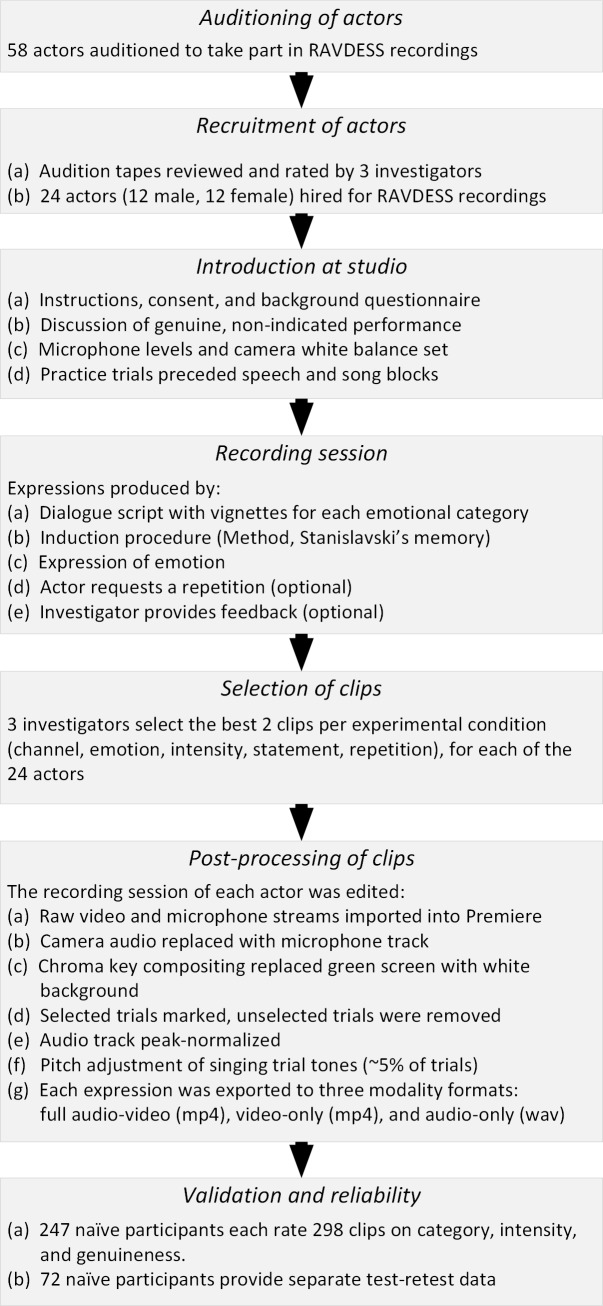
Flowchart of RAVDESS creation and validation. Flowchart illustrating the method of stimulus recording, editing, and validation.

The RAVDESS was created following the procedure outlined in Fig 1. Actors were recruited through postings made to online casting services, and contacts at the Toronto Fringe Festival. Fifty-eight actors auditioned, during which they were recorded while performing one example of each emotional condition in speech and song. Audition videos were reviewed by the first author and two research assistants (hereon, three investigators), with expressions rated in terms of accuracy, intensity, and genuineness. From this set, the 24 actors with the highest aggregate ratings were asked to return for a second recording. Actors were booked for a 4-hour recording session and were paid for their time.

Recordings took place in a professional recording studio at Ryerson University. Actors wore a black t-shirt, had minimal makeup, were clean shaven, wore contact lenses (if required), and had no distinctive jewelry. Actors were standing during all productions, with a seat provided to allow actors to rest and prepare between conditions. Microphone levels were set by having the actor produce several very angry expressions. Actors began with several practice trials of each emotional expression, and then completed all speech trials. Actors were given a 60-minute break in between blocks. Following the break, actors began with singing practice trials and then completed all singing trials. Recordings always began with speech to prevent any metrical influence of the singing condition. Trials were blocked by emotion, with low-intensity emotions followed by their very intense counterparts. This ordering allowed actors to enter and remain within the desired state for all productions of that emotional category.

A dialog script was used with all actors. A description of each emotional condition was provided. To ensure that actors understood what emotion was requested, emotional labels taken from the prototype model of emotion were used in the description [130]. A vignette describing a scenario involving that emotion was provided for each level of intensity. Actors were then given time to prepare their emotional state using their desired induction technique. For the song condition, actors were told to sing the basic notated pitches, but that they were free to vary acoustic characteristics to convey the desired emotion.

Actors could repeat a given trial until they were comfortable with their production. Actors were observed in an adjacent control room via video and audio feeds. Feedback was given if a production was felt to be ambiguous by both operators. No instruction was given as to how an emotion should be expressed. Multiple takes of each production were recorded. All takes were later reviewed by three investigators. Clips containing hand movements or gestures were removed, as were trials that contained lexical errors. After the removal of erroneous clips, the criteria for selection were productions that clearly conveyed the specified emotion and intensity through the face and the voice. The best two takes as agreed through consensus were selected for inclusion.

#### Technical information

**Fig 2:**
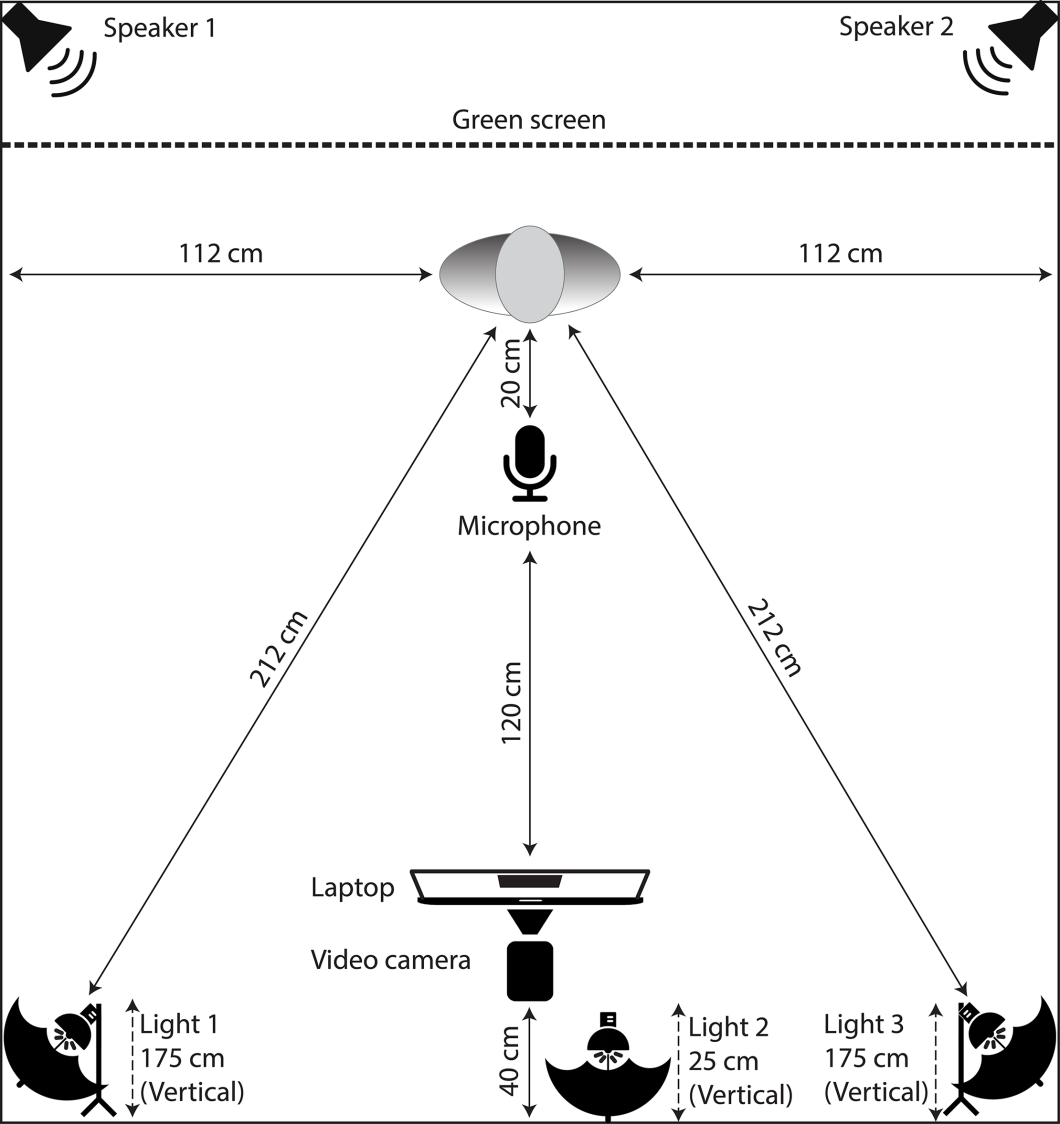
Physical setup of the recording studio. The physical layout of the recording studio used to record RAVDESS stimuli. All measurements refer to horizontal distances unless otherwise specified.

**Fig 3:**
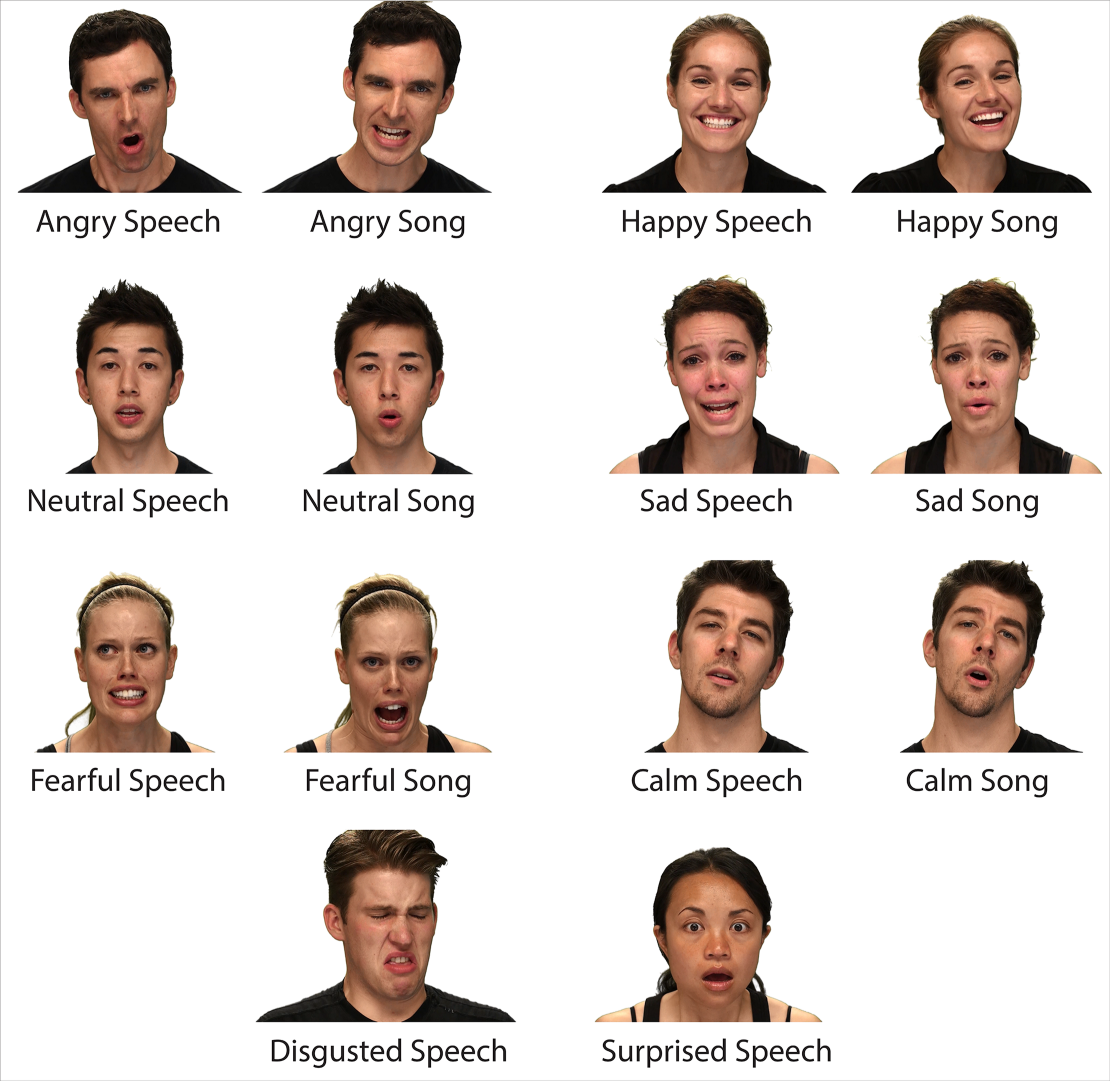
Examples of the eight RAVDESS emotions. Still frame examples of the eight emotions contained in the RAVDESS, in speech and song.

Actors were recorded individually in a professional recording studio, as illustrated in Fig 2. Actors stood in front of a Westcott digital green screen cloth and were recorded with a Sony Handycam HDR-SR11. Actors were recorded at 1080i with a scan resolution of 1920x1080 pixels at 30 fps, with files saved in AVCHD format. The camera was placed 1.4 m from the actor and zoomed to provide a fixed-width field of view of 0.5 m. Only the actor and green screen cloth were visible in the frame. The camera’s height was adjusted to ensure the actor fit within the scene, capturing their head and upper shoulders (see Fig 3). Arms and hands were not visible. Actors were illuminated by ceiling fluorescent lighting and three 28W 5200k CRI 82 bulbs, fitted in 10” reflectors with 38” white parabolic umbrellas. This setup provided full spectrum lighting while minimizing facial shadows. Voice recordings were captured by a Rode NTK vacuum tube condenser microphone, fitted with a Stedman proscreen XL pop filter, placed 20 cm from the actor. Microphone output was recorded using Pro Tools 8 and a Digidesign 003 mixing workstation, at a sampling rate of 48 kHz, 16 bit, with files saved in uncompressed wave format.

Stimuli were presented visually on a 15” Macbook Pro and auditorily over KRK Rocket 5 speakers, controlled by Matlab 2009b and the Psychophysics Toolbox [131]. Temporal accuracy of the presentation software was confirmed with the Black Box Toolkit [132]. Operator feedback was provided over speakers, with audio feeds controlled by Mackie Big Knob studio command system.

#### Post-processing and standardization of recordings

Recordings were edited using Adobe Premiere Pro CS6. The microphone stream was imported and aligned to the camera’s audio channel using predefined markers. Chroma key compositing was used to replace the green screen backdrop with a solid white background (RGB 255, 255, 255). Trials that had been selected for inclusion were marked and unwanted trials were removed from the session.

The microphone track for each actor was peak-normalized to -3 dBFS using Adobe Audition CS6. Peak normalization was chosen to retain the natural variation in loudness between emotional conditions [95, 126, 133]. The singing audio track was imported into Melodyne for pitch adjustment to ensure that the three melodies remained perceptually distinct. Intervals are perceived as “in tune” when mistuned by up to 35 cents [134, 135], and “out of tune” when mistuned by 50–100 cents [136]. Notes that were mistuned by more than 35 cents were adjusted to within ±35 cents of the target frequency.

Trials were exported using Adobe Premiere Pro CS6. Full audio-video and video-only trials were exported as MPEG-4 format (H.264, AAC) with a resolution of 1280x720 pixels at 30 fps (HD format, 720p). Audio-only files were exported as lossless wave format, at 48 kHz.

### Description of RAVDESS files

#### Experimental design

The RAVDESS contains 7356 recordings of 24 actors (12 male, 12 female). All actors produced 104 distinct vocalizations, consisting of 60 spoken utterances and 44 sung utterances. Each of the 104 vocalizations was exported to create three separate modality conditions: audio-video (face and voice), video-only (face, but no voice), and audio-only (voice, but no face). This produced 312 files per actor (104 × 3). The song recordings of one female participant were lost due to technical issues (132 files). Thus, 24 × 312–132 = 7356 files. This set is composed of 4320 speech recordings and 3036 song recordings.

Actors vocalized two distinct statements in the speech and song conditions. The two statements were each spoken with eight emotional intentions (neutral, calm, happy, sad, angry, fearful, surprise, and disgust), and sung with six emotional intentions (neutral, calm, happy, sad, angry, and fearful). All emotional conditions except neutral were vocalized at two levels of emotional intensity, normal and strong. Actors repeated each vocalization twice. The factorial design of the RAVDESS is visualized in S1 and S2 Figs.

The full design of speech trials includes: Emotional [Vocalist (12) × Gender (2) × Statement (2) × Emotion (7) × Intensity (2) × Repetition (2) × Modality (3)] + Neutral [Vocalist (12) × Gender (2) × Statement (2) × Repetition (2) × Modality (3)] = 4320 recordings. The full design of emotional and neutral trials in song was: Emotional [Vocalist (11) × Gender (2) × Statement (2) × Emotion (5) × Intensity (2) × Repetition (2) × Modality (3)] + Neutral [Vocalist (11) × Gender (2) × Statement (2) × Repetition (2) × Modality (3)] = 3036 recordings.

Still-image frames showing examples of each of the emotional expressions are illustrated in Fig 3. Full audio-video movies showing examples of each emotional expression for speech and song are presented in S1 and S2 Files respectively.

#### Filename convention

Each RAVDESS file has a unique filename. The filename consists of seven two-digit numerical identifiers, separated by hyphens (e.g., 02-01-06-01-02-01-12.mp4). Each two-digit numerical identifier defines the level of a different experimental factor. The identifiers are ordered: Modality–Channel–Emotion–Intensity–Statement–Repetition–Actor.mp4 or .wav. The numerical coding of levels is described in Table 1. For example, the filename “02-01-06-01-02-01-12.mp4” refers to: Video-only (02)–Speech (01)–Fearful (06)–Intensity normal (01)–Statement “dogs” (02)–First repetition (01)–Twelfth actor, female (12). The gender of the actor is coded by the actor’s number, where odd numbered actors are male, even numbered actors are female.

**Table 1:** Description of factor-level coding of RAVDESS filenames.

Identifier	Coding description of factor levels
Modality	01 = Audio-video, 02 = Video-only, 03 = Audio-only
Channel	01 = Speech, 02 = Song
Emotion	01 = Neutral, 02 = Calm, 03 = Happy, 04 = Sad, 05 = Angry, 06 = Fearful, 07 = Disgust, 08 = Surprised
Intensity	01 = Normal, 02 = Strong
Statement	01 = "Kids are talking by the door", 02 = "Dogs are sitting by the door"
Repetition	01 = First repetition, 02 = Second repetition
Actor	01 = First actor, …, 24 = Twenty-fourth actor

#### Download and accessibility

A main goal of the RAVDESS was to provide researchers and interested parties with a validated stimulus set that is free and accessible. To meet this goal, the RAVDESS database is released under a Creative Commons Attribution-NonCommerical-ShareAlike 4.0 license (CC BY-NA-SC 4.0). The database can be downloaded free of charge and without restriction from the open access repository Zenodo (https://doi.org/10.5281/zenodo.1188976). This manuscript and its associated validation datasets are published in PLOS ONE, an open access journal that applies the Creative Commons Attribution (CC BY) license to its articles.

### Validation of RAVDESS stimuli

#### Participants

Three hundred and nineteen undergraduate students (76% female, 24% male, mean age = 20.55 years, SD = 4.65) from Ryerson University, Toronto, Canada, participated in exchange for course credit. Raters had varied amounts of education (M = 13.97 years, SD = 2.24), private music instruction (M = 3.46 years, SD = 3.69), singing experience (M = 1.88 years, SD = 2.69), and drama experience (M = 2.35 years, SD = 2.81). All participants were fluent in English, with 75.2% identifying English as their L1. Participants identified themselves as being right-handed (91.5%), left-handed (7.84%), or ambidextrous (0.6%). No raters had taken part in stimulus creation.

#### Stimuli, apparatus, and procedure

The stimuli consisted of 7356 audio-visual (AV), video-only (VO), and audio-only (AO) recordings of emotional speech and song. Participants were tested individually in IAC double-walled sound-attenuated booths. Stimuli were presented visually on a 27” iMac, at a resolution of 2560x1440 pixels, and auditorily over Sennheiser HD 518 headphones, controlled by custom Matlab software and the Psychophysics Toolbox [131]. Volume settings were kept constant across all participants.

#### Validity task

Two hundred and forty-seven raters took part in the validity task. Raters were presented a pseudo-randomly chosen set of 298 stimuli, consisting of 174 speech and 124 song presentations. Trials were blocked and counterbalanced by Channel. Raters were seated approximately 60 cm from the computer display. In addition to verbal instruction, the following on-screen instructions were presented: “You will now be presented with recordings of people speaking and singing with different emotions. Recordings will be either: sound alone, video alone, or sound and video. After each recording, you will be asked to make three judgements: *category* of the emotion, *strength* of the emotion, and *genuineness* of the emotion. Category is the type of emotion (e.g., happy or sad). Strength is how intense the emotion was (e.g., weak or strong). Genuineness is whether you thought the person was physically, mentally, and emotionally feeling what they expressed (e.g., not genuine or very genuine).” Three practice trials preceded each Channel block, which used stimuli that were not contained in the rater’s subset.

Raters were asked to identify the category of emotion using a forced-choice response format. Speech options were: neutral, calm, happy, sad, angry, fearful, disgust, and surprise. Song options were: neutral, calm, happy, sad, angry, and fearful. The escape option “None of these are correct” was also provided [137]. Two orderings of emotion labels were used and was counterbalanced across raters. Emotion labels were listed vertically, next to a numbered box that was shaded according to Plutchik’s wheel of emotion [62]. Raters then evaluated the strength of the emotion using a 5-point Likert scale ranging from very weak (1) to very strong (5). Raters then evaluated the genuineness of the presentation using a 5-point Likert scale ranging from not genuine (1) to very genuine (5). The response rating screens are shown in S3 Fig.

Rater responses could only be provided once the feedback screen was displayed, ensuring participants viewed the entire clip. This process prevented participants from moving quickly through the task. It also eliminated any confounding effects of skipping stimuli of longer duration, as duration is known to vary consistently with emotion and intensity [66]. Raters also completed a background questionnaire. Participation in the experiment took approximately 60 minutes. All 7356 stimuli were each rated 10 times on emotional category, intensity, and genuineness, yielding 73560 ratings for each of the three measurement scales, or 220680 ratings in total.

#### Test-retest reliability task

Seventy-two raters took part in the test-retest reliability task. No participant from the validity task took part in the test-retest task. Raters began with a subset of 102 trials, consisting of 60 speech and 42 song trials. Raters were then given a 20-minute break outside the testing booth, during which time they filled out a background questionnaire. Raters then re-entered the booth and were presented the same 102 files. Trials were blocked and counterbalanced by Channel within each presentation, with different random orderings used in the first and second blocks. All other aspects of the reliability task were the same as those used in the validity task.

#### Analysis of validity task

Emotion category ratings were coded as correct (1) when the category selected by the rater matched the category that the actor had intended to express, and incorrect (0) otherwise. We use the term “proportion correct” to refer to the proportion of responses that were coded as correct, see also [29]. As proportion correct scores do not correct for response bias or false alarms, unbiased hit rates (H_u_) were also calculated [138]. Unbiased hit rates are proportion scores (0–1), and yield a smaller value than their corresponding proportion correct scores, except in the case of perfect unbiased accuracy. Unbiased hit rates were calculated as the product of Uncorrected hit rate and Differential accuracy [138]; as defined by Eq 1 where *i* is the i^th^ stimulus of interest, *n* is number of stimuli of that intended emotional category, and *N* is the total number of stimuli for that channel (speech or song).

$$UHR_{i}=\frac{\sum_{i}\left({\mathrm{Responses}}_{Intended}{=\mathrm{Responses}}_{Chosen}\right)}{\sum_{i}{\mathrm{Responses}}_{All}}\times \frac{\sum_{i}^{n}\left({\mathrm{Responses}}_{Intended}{=\mathrm{Responses}}_{Chosen}\right)}{\sum_{i}^{N}{\mathrm{Responses}}_{All}}$$ (1)

Interrater reliability is assessed with Fleiss’ kappa [139], a chance-corrected measure of inter-rater agreement for m-raters on nominal data. Kappa scores were calculated to estimate the degree of agreement between raters’ emotion category responses. These scores reflect the degree of agreement in classification over that which would be expected by chance. Kappa scores were generated for each factor of interest (reported in Table 2). These calculations involved separate n*m matrices, consisting of ‘n’ RAVDESS files and ‘m’ raters (m = 10). Category-wise kappa scores were also generated, and represent interrater reliability scores for each emotional category (reported in Table 3). It was not expected that calm and neutral expressions would be identified as distinct emotions due to their perceptual similarities. Therefore, responses of neutral or calm were accepted as correct for both neutral and calm expressions, see also [29]. Hypothesis tests were conducted during the calculation of kappa values to determine if the observed interrater agreement rates were different to those expected by chance. All tests achieved p-values < 0.001, suggesting that observed interrater agreement rates were not due to chance. For conciseness, kappa test p-values are omitted from the manuscript. Kappa values are interpreted according to the guidelines established by Landis and Koch [140], where values < 0 indicate poor agreement, 0.01–0.20 slight agreement, 0.21–0.40 fair agreement, 0.41–0.60 moderate agreement, 0.61–0.80 substantial agreement, and 0.81–1 indicate almost perfect agreement.

**Table 2:** Validity task accuracy measures across channel, modality, and intensity.

Channel	Modality	Intensity	N	Mean (SD) Proportion correct	Mean (SD) Unbiased hit rate	Mean (SD) Intensity	Mean (SD) Genu.	Kappa
Speech	AV	Normal	768	0.77 (0.23)	0.57 (0.17)	3.44 (0.51)	3.47 (0.44)	0.62
		Strong	672	0.83 (0.19)	0.62 (0.15)	4.01 (0.56)	3.56 (0.56)	0.71
	VO	Normal	768	0.70 (0.25)	0.52 (0.19)	3.40 (0.54)	3.42 (0.46)	0.53
		Strong	672	0.75 (0.25)	0.56 (0.19)	3.88 (0.60)	3.55 (0.48)	0.62
	AO	Normal	758	0.58 (0.30)	0.43 (0.22)	3.14 (0.42)	3.12 (0.41)	0.41
		Strong	672	0.67 (0.27)	0.50 (0.21)	3.71 (0.62)	3.51 (0.46)	0.52
Song	AV	Normal	552	0.77 (0.23)	0.57 (0.19)	3.37 (0.49)	3.33 (0.48)	0.61
		Strong	460	0.84 (0.20)	0.63 (0.18)	3.91 (0.58)	3.46 (0.51)	0.72
	VO	Normal	552	0.75 (0.25)	0.55 (0.21)	3.41 (0.53)	3.36 (0.46)	0.61
		Strong	460	0.79 (0.23)	0.59 (0.20)	3.89 (0.61)	3.54 (0.51)	0.67
	AO	Normal	552	0.53 (0.28)	0.39 (0.21)	3.13 (0.39)	3.24 (0.37)	0.31
		Strong	460	0.62 (0.28)	0.47 (0.23)	3.55 (0.57)	3.37 (0.40)	0.44

Description of validity ratings for spoken and sung expressions, across channel, modality, and emotional intensity (N = 247 participants, each rating 298 stimuli). AV = audio-video; VO = video only; AO = audio only. As neutral had no intensity manipulation, neutral scores were collapsed into the ‘normal’ intensity category.

**Table 3:** Validity task accuracy measures across emotion and channel.

Emotion	N	Mean (SD) Proportion correct	Mean (SD) Unbiased hit rate	Mean (SD) Intensity	Mean (SD) Genuineness	Kappa
Neutral (speech)	288	0.87 (0.14)	0.60 (0.10)	3.16 (0.44)	3.36 (0.45)	0.58
Neutral (song)	276	0.78 (0.18)	0.53 (0.12)	3.03 (0.36)	3.22 (0.40)	0.49
Calm (speech)	576	0.70 (0.24)	0.48 (0.16)	3.26 (0.41)	3.39 (0.39)	0.58
Calm (song)	552	0.63 (0.25)	0.43 (0.17)	3.24 (0.40)	3.38 (0.40)	0.49
Happy (speech)	576	0.68 (0.32)	0.49 (0.23)	3.68 (0.58)	3.51 (0.45)	0.63
Happy (song)	552	0.75 (0.29)	0.55 (0.21)	3.68 (0.59)	3.40 (0.50)	0.65
Sad (speech)	576	0.61 (0.30)	0.42 (0.21)	3.33 (0.61)	3.37 (0.45)	0.53
Sad (song)	552	0.68 (0.28)	0.43 (0.18)	3.41 (0.55)	3.34 (0.46)	0.51
Angry (speech)	576	0.81 (0.22)	0.64 (0.17)	3.96 (0.67)	3.71 (0.55)	0.67
Angry (song)	552	0.83 (0.22)	0.73 (0.19)	3.83 (0.62)	3.45 (0.51)	0.75
Fearful (speech)	576	0.71 (0.24)	0.56 (0.19)	3.76 (0.66)	3.46 (0.49)	0.60
Fearful (song)	552	0.65 (0.29)	0.51 (0.22)	3.70 (0.58)	3.37 (0.47)	0.57
Disgust (speech)	576	0.70 (0.27)	0.55 (0.21)	3.73 (0.57)	3.43 (0.46)	0.60
Surprise (speech)	552	0.72 (0.24)	0.55 (0.19)	3.53 (0.49)	3.47 (0.45)	0.60

Description of validity ratings and interrater reliability values for emotional expressions in speech and song.

Measures of inter-rater reliability were calculated for emotional intensity and genuineness scales. Separate intra-class correlations (ICC) were calculated for speech and song. ICC one-way random effects, consistency, single rater/measurement ICC(1,1) and one-way random effects, consistency, multiple raters/measurements ICC(1,k) were calculated [141]. The higher indices of ICC(2,1) and ICC(2,k) that partial out variance due to specific raters and rater × stimuli interaction were not calculated, as all raters were not presented the full set of stimuli. As one-way random-effects models generally give a smaller ICC estimate than 2-way models [142], our reliability indices are probably lower than the actual reliability of the stimulus. ICC values are reported according to the guidelines set forth recently by Koo and Li [142]. ICC values are interpreted according to the guidelines established by Cicchetti [143], where values < 0.40 indicate poor agreement, 0.40–0.59 fair agreement, 0.60–0.74 good agreement, and 0.75–1 indicate excellent agreement.

For individual stimuli, a composite “goodness” score was derived to facilitate researchers’ selection of stimuli for their research paradigm, see also [33]. Goodness values range between 0 and 10; as defined by Eq 2, where *i* is the i^th^ stimulus of interest, *P* refers to Proportion correct, *I* refers to the Intensity rating, and *G* refers to the Genuineness rating. As the neutral emotion category does not have a meaningful intensity or genuineness rating, goodness scores for these stimuli are determined only by their proportion correct scores.

$$Goodness_{i}=\left\{ \begin{array}{l} P_{i}I_{i}+P_{i}G_{i}\;\mathrm{if}\;\mathrm{Emotion}\neq \mathrm{neutral}\\ 10P_{i}\;\mathrm{otherwise} \end{array}\right.$$ (2)

Response times for emotion category, intensity, and genuineness ratings were also calculated. Response times were defined as the duration of time between the display of the response option screen and the recording of a valid keystroke by the participant. Response times that exceeded 2.2 times the inter-quartile range above the upper quartile were excluded from the data [144, 145]. That is, RT > = F_U_ + 2.2 * (F_U_—F_L_), where F_U_ and F_L_ refer to upper and lower fourth respectively. This process removed response times of unusually long duration (e.g., participant had become distracted). This process removed the response times of 4.2% of category responses (n = 3088), 2.6% of intensity responses (n = 1944), and 1.9% of genuineness responses (n = 1430).

The measures proportion correct, emotional intensity, and emotional genuineness, were examined with repeated measures analyses of variance (ANOVA). As participants were presented a subset of all stimuli, participants did not see all levels of all factors (cells). To analyze these data, responses across missing cells were collapsed to create valid factorial designs. This collapsing precluded a full-factorial ANOVA, but did permit examinations by Channel(2), Modality(3), Intensity(2), and by Emotion (7, 5) separately for each channel. Proportion scores (0–1) were arcsine transformed prior to analysis [146]. For readability, pre-transformed means are reported in the manuscript. When Mauchly’s sphericity test was significant, Greenhouse–Geisser’s correction was applied when ε < .75, and Huynh-Feldt correction when ε ≥ .75 [147]. Effect sizes are reported with partial eta-squared values. Means are accompanied by 95% confidence intervals in square brackets. Pairwise comparisons were adjusted using Bonferroni correction. All reported ANOVAs were exploratory in nature with no explicit a-priori hypotheses. As exploratory ANOVAs suffer from hidden multiplicity [148], *p*-values were corrected by controlling the false discovery rate with the Benjamini–Hochberg procedure [149]. Statistical tests were conducted in Matlab 2015a and SPSS v22.0.0.2. Reliability measures were calculated in R v3.4.3 [150] with RStudio [151], using the irr package [152], and data manipulation tools from the tidyverse package [153].

#### Analysis of test-retest reliability task

Intrarater reliability was assessed with Cohen’s kappa, a chance-corrected measure of agreement for 2 raters on nominal data. As with the validity task, kappa scores were calculated to estimate the degree of agreement between raters’ chosen emotion category responses. Separate kappa scores were generated for speech and song. These calculations involved separate n*2 matrices, consisting of ‘n’ RAVDESS files and 2 ratings. As in the validity task, responses of neutral or calm were accepted as correct for both neutral and calm expressions. Measures of intrarater reliability for emotional intensity and genuineness scales were assessed with intra-class correlations (ICC), as described above in the validity task.

## Results

### Validity task

#### Accuracy measures

There were two measures of correctness in the validity task (proportion correct and unbiased hit rate) for each stimulus, resulting in 7356 proportion correct and unbiased hit rate scores. These scores are presented individually in S1 Table, along with the measures Intensity, Genuineness, their respective response times, Goodness scores, and stimulus file duration. For conciseness, these scores are presented in Table 2 by Channel, Modality, and Intensity, along with their respective Kappa scores.

The overall proportion correct was high for speech (mean = .72, SD = .27, median = .8), as well as for song (mean = .71, SD = .27, median = .8). The overall unbiased hit rate for speech was good (mean H_u_ = .53, SD = .20, median = .57), as well as for song (mean H_u_ = .53, SD = .21, median = .55). Kappa values indicated “substantial” interrater agreement for strong expressions in speech (κ = .62, n = 2016), and song (mean κ = .61, n = 1380), and “moderate” agreement for normal expressions in speech (κ = .53, n = 2304) and song (κ = .52, n = 1656). These validity ratings are also presented in aggregate form by Emotion, for speech and song, in Table 3.

To assess the effect of presentation mode on raters’ identification accuracy, a three-way repeated measures ANOVA was conducted on raters’ proportion correct scores by Channel (2 levels: speech, song), Modality (3 levels: audio-video, video, audio), and Intensity (2 levels: normal, strong). No effect of Channel was found, F(1, 246) = 2.31, *p* = .15. A main effect of Modality was found, F (1.94, 477.53) = 941.68, p < 0.001, $\eta_{p}^{2}$ = .79. Pairwise comparisons confirmed that Audio-Video presentations *M* = .81, 95% CI [.80, .81] > Video presentations *M* = .75, [.74, .76], > Audio presentation *M* = .60, [.59, .61]. These findings are in line with existing research suggesting a face-bias in emotional recognition tasks [75, 126]. A main effect of Intensity was also found, F (1, 246) = 402.39, *p* < 0.001, $\eta_{p}^{2}$ = .62. Pairwise comparisons confirmed that stronger intensity expressions *M* = .75, [.74, .76] > normal intensity expressions *M* = .68, [.68, .69]. Comparable findings have been previously reported for face and voice recognition tasks [65, 66]. A two-way interaction of Channel × Modality was reported, F (1.92, 972.9) = 59.08, *p* < 0.001, $\eta_{p}^{2}$ = .19. Posthoc comparisons (Tukey’s HSD = .02, α = .05) confirmed that Video-Song *M* = .77, [.76, .78] > Video-Speech, *M* = .72, [.71, .73], yet Audio-Song *M* = .58, [.56, .59] < Audio-Speech *M* = .63, [.62, .64], suggesting a role in the interaction. These results are partially supported by recent findings that emotion expressed through the voice is identified less accurately in song than in speech [13]. Finally, a significant two-way interaction of Modality × Intensity was reported, F (2, 492) = 9.38, *p* < 0.001, $\eta_{p}^{2}$ = .04. Given the small effect size, we do not report posthoc comparisons.

To assess the effect of emotion on raters’ identification accuracy, separate one-way repeated measures ANOVA were conducted on raters’ proportion correct scores by Emotion in Speech (8 levels: neutral, calm, happy, sad, angry, fearful, disgust, surprise), and Song (6 levels: neutral, calm, happy, sad, angry, fearful, disgust, surprise). For speech, a main effect of Emotion was found, *F* (5.87, 1443.83) = 108.03, p < 0.001, $\eta_{p}^{2}$ = .31. Pairwise comparisons confirmed that Neutral *M* = .87, 95% CI [.85, .88] > Angry *M* = .81, [.80, .83] > Calm *M* = .70, [.68, .72] ~ Fearful *M* = .71, [.69, .73] ~ Surprise *M* = .72, [.71, .74] > Happy *M* = .68, [.67, .70] ~ Disgust *M* = .70, [.68, .71] > Sadness *M* = .61, [.59, .63]. These results are generally in line with recognition rates commonly reported in the literature [126]. For song, a main effect of Emotion was found, *F* (3.79, 932.27) = 81.33, p < 0.001, $\eta_{p}^{2}$ = .25. Pairwise comparisons confirmed that Neutral *M* = .78, 95% CI [.76, .80] ~ Angry *M* = .84, [.82, .85] > Happy *M* = .75, [.73, .76] > Sad *M* = .68, [.66, .70] > Fearful *M* = .65, [.64, .67] ~ Calm *M* = .63, [.61, .65]. To provide a more nuanced understanding of these results, proportion correct scores by Emotion, Channel, Modality, and Intensity are presented in aggregate in Table 4.

**Table 4:** Validity task mean proportion correct scores.

	Speech				Song				
	AV	VO	AO	Total	AV	VO	AO	Total	Channel Total
*Strong Intensity*									
Calm	0.72	0.58	0.75	0.68	0.66	0.59	0.64	0.63	0.66
Happy	0.84	0.89	0.44	0.72	0.93	0.90	0.50	0.78	0.75
Sad	0.81	0.77	0.62	0.73	0.85	0.83	0.51	0.73	0.73
Angry	0.94	0.92	0.91	0.92	0.93	0.90	0.86	0.89	0.91
Fearful	0.79	0.70	0.73	0.74	0.83	0.75	0.59	0.72	0.73
Disgust	0.88	0.68	0.54	0.70					0.70
Surprise	0.86	0.69	0.74	0.76					0.76
**Total for Strong Intensity**	**0.83**	**0.75**	**0.67**	**0.75**	**0.84**	**0.79**	**0.62**	**0.75**	**0.75**
*Normal Intensity*									
Calm	0.73	0.62	0.79	0.71	0.61	0.58	0.68	0.62	0.67
Happy	0.80	0.85	0.29	0.65	0.86	0.88	0.40	0.72	0.68
Sad	0.56	0.56	0.34	0.49	0.73	0.74	0.40	0.63	0.55
Angry	0.75	0.78	0.59	0.71	0.88	0.88	0.57	0.77	0.74
Fearful	0.77	0.66	0.59	0.67	0.71	0.64	0.40	0.58	0.63
Disgust	0.89	0.69	0.50	0.70					0.70
Surprise	0.82	0.61	0.62	0.68					0.68
**Total for Normal Intensity**	**0.76**	**0.68**	**0.53**	**0.66**	**0.76**	**0.75**	**0.49**	**0.66**	**0.66**
*No Intensity*									
Neutral	0.88	0.81	0.91	0.87	0.83	0.76	0.75	0.78	0.82
**Total for all intensities**	**0.80**	**0.72**	**0.62**	**0.72**	**0.80**	**0.77**	**0.57**	**0.71**	**0.71**

Validity task Mean proportion correct scores across channel, modality, emotion, and intensity, for speech and song.

Mean scores by actor for proportion correct, unbiased hit rates, intensity, genuineness, response times, goodness, and file duration are provided in S2 Table, separately for speech and song. The actors which achieved a mean proportion correct score > = 0.75 in speech were: A6, A18, A8, A2, A7, and A12. The actors which achieved a mean proportion correct score > = 0.75 in song were: A8, A7, A4, and A15. These scores represent aggregate scores only and researchers are encouraged to select files individually based on their specific requirements.

**Fig 4:**
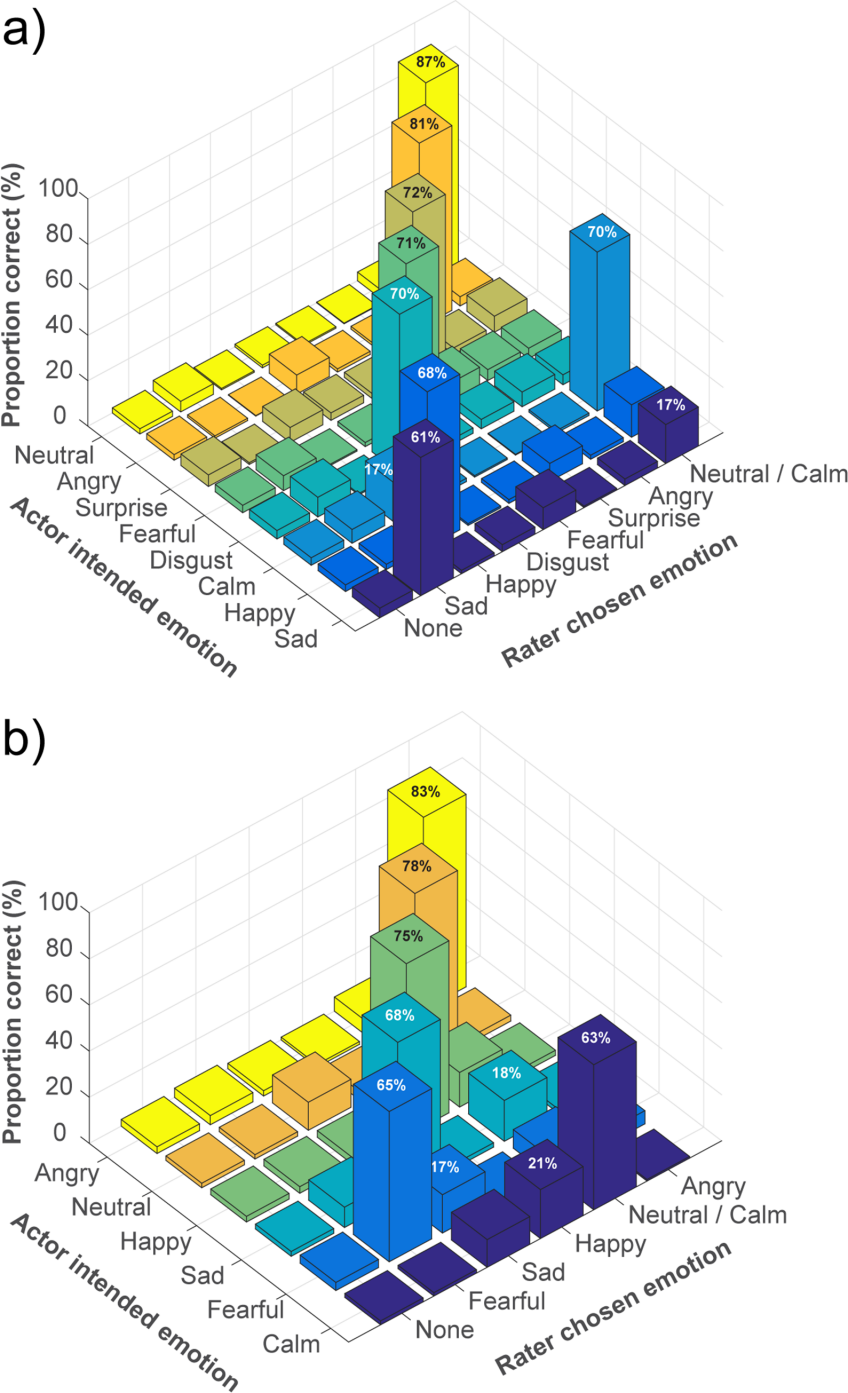
Confusion matrices of emotional validity. The confusion matrices present mean proportion correct scores for actors’ intended emotions as per rater chosen emotion labels for: (A) Speech (N = 43200 ratings), and (B) Song (N = 30360 ratings). Proportion scores that equal or exceed 15% are notated on the corresponding bar.

Confusion matrices showing the average proportion of target and non-target labels selected by raters for each intended emotional expression are presented in S3 Table. These confusion matrix data are visualized in Fig 4. The data reveal that the pattern of errors was relatively consistent across both channels.

#### Intensity and genuineness measures

Interrater reliability of the ratings provided for emotional intensity (five levels, labeled 1 to 5 from least intense to the most intense) and emotional genuineness (five levels, labeled 1 to 5 from not genuine to very genuine) were estimated with intraclass correlations, separately for speech and song, and are presented in Table 5.

**Table 5:** Validity task ICC calculations for intensity and genuineness using single- and multiple-rating, consistency-agreement, 1-way random-effects models.

Response Scale	ICC test	Value	95% Conf. Interval		F-test with True Value 0			
			Lower bound	Upper bound	Value	df1	df2	Sig
Intensity (speech)	Single (1, 1)	0.22	0.21	0.23	3.84	4319	38880	0.000
	Average (1, k)	0.74	0.73	0.75	3.84	4319	38880	0.000
Intensity (song)	Single (1, 1)	0.21	0.20	0.22	3.63	3035	27324	0.000
	Average (1, k)	0.72	0.71	0.74	3.63	3035	27324	0.000
Genuineness (speech)	Single (1, 1)	0.07	0.06	0.08	1.73	4319	38880	0.000
	Average (1, k)	0.42	0.40	0.45	1.73	4319	38880	0.000
Genuineness (song)	Single (1, 1)	0.07	0.06	0.07	1.71	3035	27324	0.000
	Average (1, k)	0.42	0.38	0.45	1.71	3035	27324	0.000

Validity task intraclass correlations of the response scales emotional intensity and genuineness, for speech and song.

Intraclass correlation single-rater values indicated “poor” agreement in speech and song for both intensity and genuineness response scales. Intraclass correlation multiple-rater values indicated “good” agreement in speech and song for both intensity and genuineness response scales. These values are comparable to those of existing sets. For intensity, the GEMEP corpus reported an average ICC(1,1) of 0.33, and ICC(1,k) of 0.9, while the Radbound Faces Database reported an ICC(1,1) of 0.20 and ICC(1,k) of 0.83. For genuineness ratings, the Radbound reported an ICC(1,1) of 0.13 and ICC(1,k) of 0.75.

To assess the effect of presentation mode on intensity ratings, a three-way repeated measures ANOVA was conducted on raters’ intensity scores by Channel (2 levels: speech, song), Modality (3 levels: audio-video, video, audio), and Intensity (2 levels: normal, strong). All statistical tests were significant. For conciseness, we only report posthoc tests for $\eta_{p}^{2}$ > .10, see also [31]. A main effect of Channel was found, *F* (1, 246) = 9.33, p = 0.003, $\eta_{p}^{2}$ = .04. Pairwise comparisons confirmed that Speech *M* = 3.6, 95% CI [3.55, 3.65] > Song *M* = 3.55, [3.50, 3.60]. A main effect of Modality was found, *F* (1.76, 433.67) = 239.86, p < 0.001, $\eta_{p}^{2}$ = .49. Pairwise comparisons confirmed that Audio-Visual presentations *M* = 3.68, [3.64, 3.733] > Video *M* = 3.65, [3.60, 3.70] > Audio *M* = 3.38, [3.30, 3.44]. A main effect of Intensity was also found, *F* (1, 246) = 1202.26, p < 0.001, $\eta_{p}^{2}$ = .83. Pairwise comparisons confirmed that Strong intensity presentations *M* = 3.83, [3.78, 3.87] > Normal intensity presentations *M* = 3.31, [3.26, 3.37]. Significant interactions were also found for Channel × Modality, F (2, 492) = 18.88, p < 0.001, $\eta_{p}^{2}$ = .07; Channel × Intensity, F (1, 246) = 16.08, p < 0.001, $\eta_{p}^{2}$ = .06; Modality × Intensity, F (2, 492) = 10.82, p < 0.001, $\eta_{p}^{2}$ = .04; and Channel ×Modality × Intensity, F (2, 492) = 11.01, p < 0.001, $\eta_{p}^{2}$ = .04.

To assess the effect of presentation mode on genuineness ratings, a three-way repeated measures ANOVA was conducted on raters’ proportion correct scores by Channel (2 levels: speech, song), Modality (3 levels: audio-video, video, audio), and Intensity (2 levels: normal, strong). A main effect of Channel was found, *F* (1, 246) = 22.35, p < 0.001, $\eta_{p}^{2}$ = .08. Pairwise comparisons confirmed that Speech *M* = 3.47, 95% CI [3.4, 3.54] > Song *M* = 3.38, [3.31, 3.45]. A main effect of Modality was found, *F* (1.81, 444.68) = 19.89, p < 0.001, $\eta_{p}^{2}$ = .08. Pairwise comparisons confirmed that Video *M* = 3.47, [3.40, 3.54] ~ Audio-Video *M* = 3.46, [3.38, 3.53] > Audio *M* = 3.36, [3.29, 3.42]. A main effect of Intensity was found, *F* (1, 246) = 47.0, p < 0.001, $\eta_{p}^{2}$ = .16. Pairwise comparisons confirmed that Strong intensity presentations *M* = 3.50, [3.42, 3.57] > Normal intensity presentations *M* = 3.36, [3.29, 3.42]. Significant interactions were also found for Channel × Modality, F (2, 492) = 14.38, p < 0.001, $\eta_{p}^{2}$ = .06 and Channel × Modality × Intensity, F (2, 492) = 4.71, p = 0.01, $\eta_{p}^{2}$ = .02.

### Test-retest reliability task

The overall proportion correct for speech at Time 1 was high (mean = .70, SD = .46), and was comparable to accuracy rates at Time 2 (mean = .72, SD = .45). The overall proportion correct for song at Time 1 was also high (mean = .71, SD = .46), and was comparable to accuracy rates at Time 2 (mean = .71, SD = .45). Intrarater reliability scores were calculated to quantify test—retest reliability of the stimuli. Kappa values indicated “substantial” intrarater reliability for strong expressions in speech (κ = .76, n = 2016), and song (mean κ = .77, n = 1380), and “substantial” reliability for normal expressions in speech (κ = .70, n = 2304) and song (κ = .68, n = 1656). Category-wise values are reported by Emotion, for speech and song, in Table 6.

**Table 6:** Test-retest task intrarater reliability ratings by emotion and channel.

Emotion	Mean Proportion correct Time 1 (SD)	Mean Proportion correct Time 2 (SD)	Kappa
Neutral (speech)	0.85 (0.36)	0.89 (0.31)	0.75
Neutral (song)	0.78 (0.42)	0.82 (0.39)	0.67
Calm (speech)	0.73 (0.45)	0.72 (0.45)	0.75
Calm (song)	0.64 (0.48)	0.62 (0.49)	0.67
Happy (speech)	0.67 (0.47)	0.67 (0.47)	0.77
Happy (song)	0.73 (0.44)	0.75 (0.44)	0.79
Sad (speech)	0.62 (0.49)	0.61 (0.49)	0.73
Sad (song)	0.69 (0.46)	0.67 (0.47)	0.70
Angry (speech)	0.77 (0.42)	0.80 (0.40)	0.77
Angry (song)	0.82 (0.38)	0.83 (0.37)	0.83
Fearful (speech)	0.71 (0.45)	0.73 (0.44)	0.75
Fearful (song)	0.62 (0.49)	0.64 (0.48)	0.72
Disgust (speech)	0.68 (0.47)	0.71 (0.45)	0.73
Surprise (speech)	0.68 (0.44)	0.73 (0.44)	0.73

Ratings from the test-retest intrarater reliability task across emotions, in speech and song (N = 72 participants, each rating 102 stimuli twice).

Intrarater reliability of the ratings provided for emotional intensity and emotional genuineness at Time 1 and Time 2 were estimated with intraclass correlations, separately for speech and song, and are presented in Table 7.

**Table 7:** Test-retest task intrarater ICC calculations for intensity and genuineness using single- and multiple-rating, consistency-agreement, 1-way random-effects models.

Response Scale	ICC test	Value	95% Conf. Interval		F-test with True Value 0			
			Lower bound	Upper bound	Value	df1	df2	Sig
Intensity (speech)	Single (1, 1)	0.46	0.44	0.49	2.71	4319	4320	0.000
	Average (1, k)	0.63	0.61	0.65	2.71	4319	4320	0.000
Intensity (song)	Single (1, 1)	0.46	0.43	0.49	2.70	3035	3036	0.000
	Average (1, k)	0.63	0.60	0.66	2.70	3035	3036	0.000
Genuineness (speech)	Single (1, 1)	0.42	0.39	0.44	2.42	4319	4320	0.000
	Average (1, k)	0.59	0.56	0.61	2.42	4319	4320	0.000
Genuineness (song)	Single (1, 1)	0.43	0.40	0.45	2.48	3035	3036	0.000
	Average (1, k)	0.60	0.57	0.62	2.48	3035	3036	0.000

Intrarater intraclass correlation single-rater values indicated “fair” agreement in speech and song for both intensity and genuineness response scales. Intraclass correlation multiple-rater values indicated “fair” to “good” agreement in speech and song for both intensity and genuineness response scales.

## Discussion

In this paper, we described the construction and validation of the RAVDESS, a set of emotional expressions that are dynamic and multimodal. The RAVDESS has several important features that lend itself for use by scientists, engineers, and clinicians: it is large in number, it contains visual and auditory depictions of spoken and sung expressions, it consists of professional actors from North America, it has a variety of emotional expressions at two levels of emotional intensity, and it is made freely available under a Creative Commons non-commercial license.

Validation of the RAVDESS was performed with 247 raters from North America. Validity referred to the accuracy with which participants correctly identified the actors’ intended emotions. We examined proportion correct scores, as is commonly reported in the literature. Overall scores were high, achieving 80% for audio-video, 75% for video-only, and 60% for audio-only. These scores are comparable to the CREMA-D, the only other validated English database of audio-visual vocal emotion [52], which achieved 64%, 58%, and 41% respectively for the three modalities, and those of the GEMEP [57], consisting of French audio-visual vocal expressions, which achieved 73%, 59%, and 44% respectively for the three modalities. Audio-only productions also fared well against batteries of affective vocal productions, including the Montreal affective voices [50] at 69%, Portuguese sentences [117] at 75%, and German sentences [99] at 85% (calculated from files received from the author, Dr. Burkhardt, through personal communication). As proportion correct scores do not correct for false alarms, unbiased hit rate were also reported, as were Fleiss’ kappa, a chance-corrected measure of interrater reliability. According to the guidelines provided by Landis and Koch [140], strong expressions of emotion fell within the substantial range of inter-rater agreement with a mean kappa of 0.61, while normal intensity expressions fell within the moderate range of inter-rater reliability with a mean kappa of 0.53.

Test-retest reliability of the RAVDESS was assessed with an additional 72 raters from North America. Reliability referred to the likelihood of participants selecting the same emotional category for a given stimulus presented twice. Cohen’s kappa scores were used to quantify the degree of intrarater agreement. Test-retest reliability was high, with a mean kappa of 0.73, falling well within substantial range of intrarater agreement. We are not aware of any other database that has provided test-retest kappa scores. Collectively, these results confirm that the RAVDESS has good validity and test-retest reliability.

Validity measures revealed variations in accuracy across emotional categories and presentation modalities. These variations are common in studies of emotional perception and reflect the nature of emotion as a complex form of communication, one that is strongly affected by the mode of presentation [7, 75, 127]. Strong intensity audiovisual displays were identified with 83% accuracy, which is comparable to the mean accuracy rates reported for the Pictures of Facial Affect at 88% [21], JACFEE at 74% [154], and NimStim at 79% [29]. These same displays presented in audio-only achieved 65% accuracy. The effect of modality appeared to vary with emotion; disgust and happiness achieved 88% and 84% accuracy in audio-visual speech yet 54% and 44% in audio-only speech; while anger achieved over 90% in both modalities. A recent review of multimodal emotional perception similarly found a face-bias for happiness and disgust, but not anger [127].

Sung expressions of emotion performed comparably with spoken expressions, achieving 71% and 72% respectively. The inclusion of a lexically-matched set of emotional song is an important distinguishing feature of the RAVDESS. To our knowledge, the RAVDESS is the only validated set of emotional song, and is one of only a handful of validated sets of musical emotion [155, 156]. The scope of the song set, at 3036 files, is significantly larger than existing sets, which contain fewer than 250 clips. The RAVDESS is the only set that includes audio-visual and video-only displays of musical emotion. There is significant research highlighting the importance of visual information in the expression of musical emotion [157–159] and the coordination of music performance [160]. The RAVDESS may therefore be of interest to researchers in music cognition and computer music performance.

The intensity of actors’ productions had a large effect on participant ratings. Strong intensity productions were identified more accurately, were rated as more emotionally intense, and rated as more genuine that normal intensity productions. These results are in line with research which has shown that strongly intense displays are identified more accurately in faces and voices [53, 65, 66]. Production studies have revealed differences in the facial and vocal expressions of intense emotions. Facial expressions with increased muscle contraction are rated as more emotionally intense [65]. Head movements of vocalists exhibit larger and faster movements, and greater rotational turning when expressing intense emotions [161]. Acoustic profiles of the voice also show clear differences in emotional intensity [66, 162]. These findings suggest that intense expressions, like those in the RAVDESS, have facial and vocal features that are more readily identified than their less intense counterparts.

Validation measures revealed a pattern of confusions between several emotion categories. Calm was misidentified as happy for 19% of responses, sad as neutral or calm at 17%, and happy as neutral or calm at 14%. Previous research has found that neutral productions convey a mildly negative emotional valence [71]. Raters misidentification of sadness with neutral/calm support this finding. Calm was included as a second baseline expression to convey a mild, positively valenced emotion. Misidentification rates suggests that raters confused happy with the mildly positively valenced calm expressions.

Ratings of emotional intensity and genuineness were also reported. Both inter-rater and intrarater reliability of these scales was assessed with intraclass correlations. According to the guidelines provided by Cicchetti [143], inter-rater single-measure ratings of intensity and genuineness fell within the poor range of reliability, and good-to-fair range respectively for average-measure ratings. These results suggest that there was little-to-moderate consistency between raters in their evaluations of intensity and genuineness. Interestingly, test-retest intrarater reliability fell within the fair range for single-measures, and good-to-fair range respectively for average-measure ratings. These results suggest that ratings of intensity and genuineness were more consistent in the context of test-retest than in the context of between raters. That is, raters were more consistent in their own ratings across multiple presentations, but that these ratings were more variable between raters. Collectively, this suggests that while intensity had a strong effect on raters’ accuracy of emotional identification, the emotional properties of intensity and genuineness were not identified consistently by raters. To our knowledge, there has been no investigation assessing the accuracy with which emotional intensity or genuineness can be identified, as these measures are typically assessed using a continuous Likert-scale response paradigm. Thus, it is unclear if the reported reliability values are a function of the RAVDESS stimuli or a more general property of these emotional concepts. This topic warrants further study. Regardless, investigators should interpret measures of intensity and genuineness with caution when selecting appropriate stimuli.

The RAVDESS included a set of six basic emotions that are thought to be culturally universal. This decision was based partly on the design goal of providing a set of emotions with high discriminability. A criticism of universal emotions is that there are few positively-valenced states [112, 163, 164]. Several sets have sought to overcome this issue [50, 57, 165]. Two of these sets developed non-verbal utterances, including pleasure [50], and pleasure, triumph, amusement, relief [165]. While these audio-only, non-verbal utterances were accurately identified, to our knowledge there has been no validation of these states in facial-only or facial-verbal modalities. Recent research also suggests that the acoustic expression of these states may not be culturally universal [166]. We chose not to include these states as face-and-voice and face-only are both integral modalities of expression in the RAVDESS. The GEMEP also included a broader range of positive emotions (pleasure, elated, joy, pride, amusement, relief, and interest). However, most of these states achieved recognition rates at or below 40%. As the authors note, empirical evidence on their expressive characteristics is scarce. As a primary goal of the RAVDESS was to provide emotions with high discriminability, we opted not to include additional “positive” emotional states.

The construction and validation of the RAVDESS used aspects of both the discrete and continuous theories of emotion. The division of emotions into distinct categories with verbal labels (e.g., happy, sad) is grounded in discrete emotion theory. Emotions were also produced and rated in terms of their emotional intensity–a continuous scale which draws from dimensional models of emotion. Dimensional models began with the works of Spencer [167] and Wundt [168], and classify emotions as existing within a multidimensional space, generally defined by the orthogonal dimensions of arousal and valence [64, 169–174]. Perceptual ratings of emotional stimuli often involve ratings along the dimensions of arousal and valence [105]. An important avenue for future work with the RAVDESS will be to provide dimensional ratings of arousal and valence.

Stimuli were validated using a forced-choice emotion response format. A criticism of forced-choice emotion paradigms is that they can bias the participant towards a hypothesis, leading to artificially forced agreement [113]. To address this criticism, our response paradigm included the “None of these are correct” option, proposed by Frank and Stennett [137]. Participants selected this option less than 3% of the time (see S3 Table), providing further support for the contention that RAVDESS actors provided clear exemplars of emotion.

The RAVDESS was constructed using induced expressions of emotion. These expressions were elicited using techniques the actors had been trained in, including method acting or emotional memory techniques. This form of elicitation has been used successfully in previous studies with the goal of producing more authentic displays [57, 125, 161, 162]. Other methods of inducing an emotional state including presentation of films or music, mental imagery methods, or stressor scenarios. However, these procedures can produce relatively weak effects, and there may be uncertainty as to the emotion that was elicited [175, 176]. For these reasons, we opted for induction techniques that our actors had used throughout their careers.

The use of induced emotional expressions contrasts with sets that use naturally occurring spontaneous or “in the wild” expressions. In these sets, recordings of individuals in real-life situations are taken from a variety of sources, such as television, clinical interviews, lost baggage offices, and online video streaming services [177]. Both approaches have strengths and weaknesses. A criticism of induced expressions is that they can be exaggerated, leading to inflated rates of observer agreement relative to spontaneous displays [75, 178–180]. There may also be fewer individuals in induced sets, which commonly use a within-subjects design. This contrasts with spontaneous sets that may have hundreds or thousands of different individuals in a between-subjects format. However, induced expressions offer several important advantages over spontaneous expressions. First, experimenters have confidence in the emotion being expressed. This contrasts with naturalistic recordings in which the emotional category of the expression is labelled after the fact by the experimenter or participant ratings. This labelling procedure raises serious concerns about the reliability of the assigned categories, as well whether the expressions reflect truly natural emotions [127, 181]. Second, induced expressions are intended to convey a single emotional category, for example “happy” or “sad”. Naturalistic recordings however are often given mixed labels by raters, with all but a few given a single clear category [182]. Finally, induced sets maintain good experimental control where actors can be recorded expressing every emotional category, using repeated lexical material, while environmental aspects including lighting, clothing, recording equipment, and background setting can remain constant. This contrasts with naturalistic sets where individuals appear in only one or two clips, and the recording situation, material, and quality can vary substantially [126].

The RAVDESS is large in scope, containing 7356 validated presentations of emotion. During construction, several sets removed stimuli that were identified at or below defined accuracy levels [50, 183], while others produced core sets containing “optimal” stimuli [57, 119]. In this initial iteration of the RAVDESS, we chose to include the full corpus of recordings. These recordings and their ratings provide researchers with a rich dataset of highly accurate, mixed, and inaccurate expressions of emotion. A significant body of literature has been dedicated to identifying what features lead to an accurate emotional expression. However, much can be learned from why particular expressions are conveyed inaccurately. These recordings provide researchers with a large data set from which to examine questions related to both the accuracy and inaccuracy of emotional expressions.

There are several shortcomings of the RAVDESS. Firstly, the scope of the database precluded the use of a fully within-subjects rating methodology. The validity task presented a subset of 298 clips to each rater. We opted for this design as it provided greater representative statistical variance over the use of a limited pool of fully within-subjects raters–an approach that has been criticized [113]. This design choice however limited the range of statistical tests that could be reported. Despite this, the reported measures provided evidence of high validity and reliability. Relatedly, each recording was only rated 10 times. While several sets have used similar numbers of ratings [32, 52], this may not be sufficient for certain paradigms. Another shortcoming was the exclusion of “self-conscious” emotions, such as shame, pride, and embarrassment [184–186]. We chose not to include these expressions as there is limited evidence that these states can be conveyed effectively through vocal communication, as prior research has focused on facial expressions and body postures. As with the exclusion of surprise and disgust in the song corpus, we opted to include emotions that are known to be reliably and accurately expressed through vocal communication. Another limitation was the use of trained investigators for the review and selection of actors’ raw productions, rather than using large-scale perceptual tests. This decision reflected the need to remove problematic stimuli (e.g., presence of hand movements and gestures, lexical errors, microphone peaking and pops), and to select the clearest exemplars of emotion. The use of expert investigators for an initial review of raw productions during recording or post-recording is common in emotion sets [32, 33, 57, 58, 76, 119, 156, 165, 187–190]. However, a consequence of this procedure may have been a selection bias driven by investigators’ prior expectations for prototypical expressions. A final limitation was the inclusion of only two statements, limiting the lexical variability of the database. While increased lexical variability would have been beneficial, we chose to prioritize diversity in actors, emotions, and intensities, while matching speech-and-song productions. As adding a third statement would have increased the size of the database by 3678 files, and required an additional 125 raters, we opted to use only two statements in the RAVDESS.

## Conclusion

In this paper, we presented the Ryerson Audio-Visual Database of Emotional Speech and Song, a set of multimodal, dynamic expressions of basic emotions. The RAVDESS is one of only two databases of audiovisual vocal expressions presented in North American English. The set consists of a large number unique speech and song recordings, each available in audio-visual, video-only, and audio-only high-definition formats. Participant testing involving untrained research participants revealed high rates of emotional validity and test-retest reliability. We believe this set will be of interest to a wide variety of researchers and engineers. The RAVDESS is made freely available under a Creative Commons non-commercial license, and can be downloaded at https://doi.org/10.5281/zenodo.1188976.

## Supporting information

S1 Fig Tree diagram of experimental design of speech corpus. Breakdown of experimental factor-levels and number of recordings per factor-level. Square brackets report [number of files for that specific internal-node, and total number of files for that factor-level]. For example, female-vocalist-1 has 60 face-video recordings, while there are 1440 face-video recordings in the set. A double outlined box indicates a leaf-node.
(TIF)

S2 Fig Tree diagram of experimental design of song corpus. Breakdown of experimental factor-levels and number of recordings per factor-level. Square brackets report [number of files for that specific internal-node, and total number of files for that factor-level]. For example, female-vocalist-1 has 44 face-video recordings, while there are 1012 face-video recordings in the set. A double outlined box indicates a leaf-node. Note, the song corpus contains 11 females and 12 males, and differs to the speech corpus which is matched on gender (12 each).
(TIF)

S3 Fig Response task option screens. Response option screens presented to participants during the validity and reliability tasks, showing: (a) Emotion category (b) Emotional intensity (c) Genuineness.
(TIF)

S1 Table Validity task measures for all stimuli. Proportion correct scores, unbiased hit rates, intensity ratings, genuineness ratings, their respective response times, and goodness scores, for all 7356 RAVDESS stimuli.
(XLSX)

S2 Table Validity task measures summarized by actor. Mean scores by actor for proportion correct, unbiased hit rates, intensity, genuineness, response times, goodness, and file duration, separately for speech and song.
(XLSX)

S3 Table Confusion matrices of proportion correct measures. Confusion matrices showing the average proportion of target and non-target labels selected by raters for each intended emotional expression, for speech and song.
(XLSX)

S4 Table Raw test-retest response data for all stimuli. Raw response data from the test-retest reliability task for all 7356 stimuli. Includes rater identified emotional category (neutral 1, calm 2, happy 3, sad 4, angry 5, fearful 6, surprise 7, disgust 8, none 9), and coded raw accuracy (incorrect 0, correct 1), for presentations at Time 1 and Time 2.
(XLSX)

S5 Table Raw validity response data for all stimuli. Raw response data from the validity task for all 7356 stimuli. Includes rater identified emotional category (neutral 1, calm 2, happy 3, sad 4, angry 5, fearful 6, surprise 7, disgust 8, none 9), coded raw accuracy (incorrect 0, correct 1), emotional intensity (very weak 1, to very strong 5), and emotional genuineness (not genuine 1, to very genuine 5).
(XLSX)

S1 File Audiovisual examples of RAVDESS speech stimuli. Movie file presenting strong intensity displays of eight speech emotions: neutral, calm, happy, sad, angry, fearful, disgust, and surprise.
(MP4)

S2 File Audiovisual examples of RAVDESS song stimuli. Movie file presenting strong intensity displays of six speech emotions: neutral, calm, happy, sad, angry, and fearful.
(MP4)
