# Supplementary figures and images for: The Ryerson Audio-Visual Database of Emotional Speech and Song (RAVDESS): A dynamic, multimodal set of facial and vocal expressions in North American English
Source: PLoS One. 2018 May 16;13(5):e0196391. doi: 10.1371/journal.pone.0196391 (PMC5955500; doi:10.1371/journal.pone.0196391)

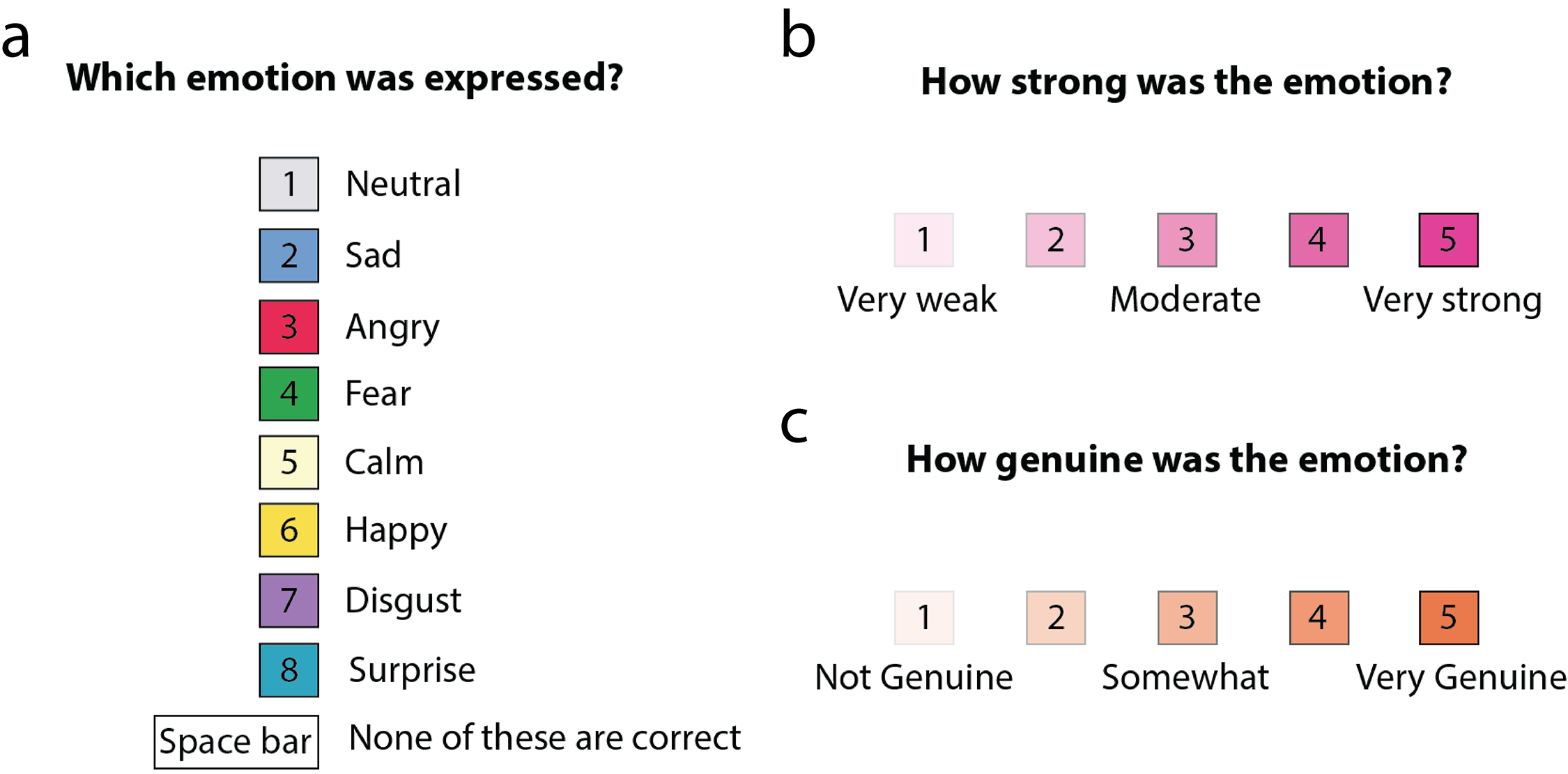

Supplement: S3 Fig — Response option screens presented to participants during the validity and reliability tasks, showing: (a) Emotion category (b) Emotional intensity (c) Genuineness. (TIF) [file pone.0196391.s003.tif]
